# Toll-like receptor-4 mediates cigarette smoke-induced cytokine production by human macrophages

**DOI:** 10.1186/1465-9921-7-66

**Published:** 2006-04-19

**Authors:** Khalil Karimi, Hadi Sarir, Esmaeil Mortaz, Joost J Smit, Hossein Hosseini, Sjef J De Kimpe, Frans P Nijkamp, Gert Folkerts

**Affiliations:** 1 Department of Pharmacology and Pathophysiology, Utrecht Institute for Pharmaceutical Sciences, Utrecht University, PO BOX 80.082, 3508 TB Utrecht, The Netherlands; 2 Department of Pathology and Molecular Medicine, Centre for Gene Therapeutics, McMaster University, 1200 Main St W, Hamilton, L8N 3Z5, Ontario, Canada

## Abstract

**Background:**

The major risk factor for the development of COPD is cigarette smoking. Smoking causes activation of resident cells and the recruitment of inflammatory cells into the lungs, which leads to release of pro-inflammatory cytokines, chemotactic factors, oxygen radicals and proteases. In the present study evidence is found for a new cellular mechanism that refers to a link between smoking and inflammation in lungs.

**Methods:**

Employing human monocyte-derived macrophages, different techniques including FACS analysis, Cytometric Bead Array Assay and ELISA were achieved to evaluate the effects of CS on pro-inflammatory cytokine secretion including IL-8. Then, Toll-like receptor neutralization was performed to study the involvement of Toll-like receptor-4 in IL-8 production. Finally, signaling pathways in macrophages after exposure to CS medium were investigated performing ELISA and Western analysis.

**Results:**

We demonstrate that especially human monocytes are sensitive to produce IL-8 upon cigarette smoke stimulation compared to lymphocytes or neutrophils. Moreover, monocyte-derived macrophages produce high amounts of the cytokine. The IL-8 production is dependent on Toll-like receptor 4 stimulation and LPS is not involved. Further research resolved the cellular mechanism by which cigarette smoke induces cytokine production in monocyte-derived macrophages. Cigarette smoke causes subsequently a concentration-dependent phosphorylation of IRAK and degradation of TRAF6. Moreover, IκBα was phosphorylated which suggests involvement of NF-κB. In addition, NFκB -inhibitor blocked cigarette smoke-induced IL-8 production.

**Conclusion:**

These findings link cigarette smoke to inflammation and lead to new insights/therapeutic strategies in the pathogenesis of lung emphysema.

## Background

Chronic Obstructive Pulmonary Disease (COPD) is a multicomponent disease [1,2] and is associated with an airway inflammatory profile consisting mainly of an increased number of CD8^+^T cells, macrophages, and neutrophils [3-5]. The major risk factor for the development of COPD is cigarette smoking. Smoking causes activation of resident cells and the recruitment of inflammatory cells into the lungs, which leads to release of pro-inflammatory cytokines, chemotactic factors, oxygen radicals and proteases [6]. Airway inflammation in COPD involves inflammatory mediators such as interleukin (IL)-8 and tumor necrosis factor (TNF)-α which are generally considered to be important mediators in neutrophil recruitment [7-9]. Many observations suggested macrophages to be the orchestrators of chronic response and tissue destructions in COPD [10-12]. For instance, macrophages in broncho alveolar lavage (BAL) from asymptomatic smokers and patients with COPD are higher than in BAL from nonsmokers [13]. Macrophages produce cytokines including IL-8 and the levels of IL-8 in induced sputum are correlated with the extent of inflammation and severity of COPD [14]. In alveolar cells, cigarette smoke (CS) constituents induce mRNA expression of inflammatory cytokines like IL-1α, IL-1β, and IL-6 [15]. Moreover, cultured human bronchial epithelial cells [16] and alveolar macrophages [17] release IL-8 in response to CS medium prepared by bubbling smoke through cell culture medium.

The Toll-like receptors (TLRs) are an evolutionarily conserved family of cell surface molecules which participate in innate immune response[18]. Among TLR family the best described and most studied is TLR2 and TLR4. TLR2 and TLR4 are shown to be expressed maximally in CD14 positive mononuclear cells within fractionated peripheral blood leukocytes [19]. Activation of macrophages through the TLR4 signal transduction pathway leads to nuclear factor (NF)-κB activation and the production of pro-inflammatory mediators like IL-8 [20]. Since CS may provide many potential inflammatory stimuli and the role of TLR proteins in inflammatory airway diseases, such as asthma and allergy is being intensively studied [21], we hypothesized that CS medium may contribute to the pathogenesis of COPD by stimulation of macrophages through ligation of TLRs. To examine the objection, firstly, the effects of CS on pro-inflammatory cytokine secretion including IL-8 were evaluated. Then, the involvement of TLR2 and TLR4 in IL-8 production was studied and, finally, signaling pathways in human monocyte-derived macrophages after exposure to CS medium were investigated. The findings explain the possible mechanisms behind the initial inflammatory process in lungs.

## Methods

### Isolation of PBMC and culture of human monocyte-derived macrophages

Peripheral blood mononuclear cells (PBMC) were separated [22] by density gradient centrifugation (Pharmacia Biotech, Uppsala, Sweden) of buffy coats obtained from normal blood donors. Thereafter, neutrophils were prepared [23] by centrifugation on a Percoll density gradient (purity 90%). The remained cells used for preparation of lymphocyte fraction by centrifugation on a Percoll density gradient (purity 85%). Human blood monocytes were obtained using RosetteSep™ (Stem cell Technologies) according to manufacturer's instructions. Briefly, fresh blood was incubated with RosetteSep™ cocktail at room temperature followed by Ficoll-Paque gradient centrifugation (Life Technologies, Cergy Pontoise, France). The enriched monocytes were collected from the Ficoll:plasma interface and purity was assessed by FACS analysis using a FITC-labeled anti-CD14 mAb (95%). Macrophages were obtained by culturing monocytes for 5 days in medium containing 2.5 ng/ml GM-CSF and 25 ng/ml M-CSF (R&D), as described before [24].

### CS medium preparation

CS medium was prepared as described before [25,26]. Briefly, a smoking machine (Teague Enterprises, Davis, CA, USA) was used to direct main and side stream smoke from one cigarette through 5 ml culture medium (RPMI without phenol red). Hereafter, absorbance was measured spectrophotometrically and the media was standardized to a standard curve of CS medium concentration against absorbance at 320 nm. This concentration was serially diluted with untreated media and applied to the cells. Freshly prepared CS medium was used in all experiments. Nontoxic concentrations of CS medium were detected performing different toxicological assays (SRB, WST-1, and LDH) and FACS analysis (annexin-V and 7-AAD staining).

### Quantification of human cytokines

Cells were plated at a density of 5 × 10^5 ^cells/ml in 96-well cell culture plates and stimulated with different concentrations of CS medium or LPS (as positive controls) for overnight. In defined experiments, cells were pretreated with SB 203580 (5 μM) or curcumin (25 μM) (both from Calbiochem) for 30 min before stimulation with CS medium. Hereafter, supernatants were collected and stored at -20 C prior to cytokine quantification. Commercially available enzyme-linked immunosorbent assay (ELISA) kits (R&D systems) or Cytometric Beads Array (CBA) kits (BD Biosciences) were used to quantify cytokine secretion according to the manufacturer's instructions. For CBA, analyses were run on a FACSCalibur^®^. Quadruplicate samples were mixed and used as a sample for the assay.

### Intracellular cytokine staining

1 × 10^6 ^cells/ml were stimulated by different concentrations of CS medium and were incubated for 5 h in the presence of the protein transport inhibitor GolgiStop™ (Pharmingen, San Diego, CA, USA). Next, cells were stained for surface antigens prior to fixation by a 4% paraformaldehyde solution. After 24 hours, the cells were permeabilized in Cytofix/Cytoperm™ solution and stained for intracellular cytokine expression (all from Pharmingen, San Diego, CA, USA).

### Anti-TLR neutralization of cytokine production

Cells were incubated with anti-human TLR2 (clone TL2.1) or mouse IgG2a isotype control (20 μg/ml), for 30 min at room temperature or with anti-human TLR4 (clone HTA125) or mouse IgG2a isotype control (20 μg/ml), (all from eBioscience, CA, USA) for one hr at 37°C. Hereafter, cells were stimulated with different concentrations of CS medium or LPS or PMA/ionomycin (Sigma) and incubated overnight. Supernatants were collected and stored at -20°C prior to cytokine quantifications.

### Western analysis

Treated cells were lysed in ice-cold buffer (containing 50 mM Tris (pH 8.0), 110 mM NaCl, 5 mM EDTA, 1% Triton X-100, and 100 μg/ml PMSF) and protein concentrations were determined performing Bradford assay. Whole cell lysates were boiled in equal volumes of loading buffer (125 mM Tris·HCl, pH 6.8, 4% SDS, 20% glycerol, and 10%2-mercaptoethanol) and 50 μg of proteins loaded per lane on an 8–16% Tris-glycine gradient gel (Novex, San Diego, CA). Proteins were electrophoretically separated and transferred to nitrocellulose membranes (Novex) using the Novex Xcell Mini-Gel system. For immunoblotting, membranes were blocked with 10% non-fat dried milk in Tris-buffered saline (TBS). Primary antibodies against human IκBα, phospho IκBα, human IRAK, and human TRAF (Santa Cruz Biotechnology) and appropriate peroxidase-conjugated secondary antibodies (Calbiochem, La Jolla, CA) were applied. Blots were incubated in commercial enhanced chemiluminescence reagents (ECL; Amersham, Buckinghamshire, England), and exposed to photographic film. Films were analyzed on a GS7–10 Calibrated Imaging Densitometer equiped with Quantity One v. 4.0.3 software (Bio-Rad Laboratories, Veenendaal, The Netherlands).

### Preparation of cytoplasmic and nuclear extracts

Cells were washed twice with PBS and allowed to equilibrate for 5 min in the ice-cold cytoplasmic extraction reagent (Pierce) containing protease inhibitors (MiniTM protease inhibitors, cocktail). Thereafter, cells were lysed and the supernatant (the cytoplasmic extracts) were collected and frozen at -70°C. To obtain the nuclear extracts, the pellets were suspended in the nuclear extraction buffer containing protease inhibitors. The solution was clarified by centrifugation at 14,000 g for 5 min after a vigorous mixing and 10 min incubation on ice. The supernatant (nuclear extracts) was collected and stored at -70°C. Protein concentrations were determined using a BCA protein assay kit (Pierce). The lysates (30 mg) from cytoplasmic or nuclear fractions were subjected to SDS/PAGE [10% (w/v) gel] for detection of P65 or actin expression.

### Statistic analysis

Unpaired Student's t tests (two-tailed) were performed using GraphPad PRISM software (version 4.00 for Windows; GraphPad, San Diego, CA). A value of p < 0.05 was considered significant. The error bars in the bar graphs show the SEM.

## Results

### Human monocyte-derived macrophages produce IL-8 in response to CS medium

**Figure 1:**
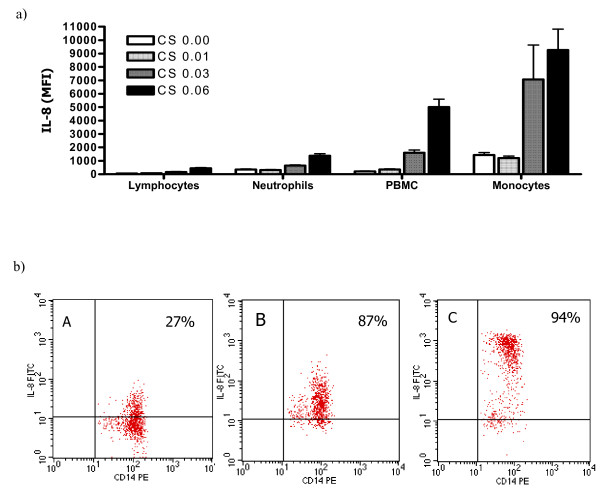
In PBMC, human monocytes are the major source of IL-8 production in response to CS medium stimulation. a) Different fractions of peripheral blood cells were isolated and stimulated with different concentrations of CS medium. Cytometric Beads Array assay was performed. Data represent the mean ± SD of two experiments conducted with different PBMC preparations. b). Human PBMC were left in culture medium (A) or were stimulated for 6 hours with CS medium (B) or LPS (C) in the presence of GolgiStop™. The cells were stained for surface marker CD14 and intracellular IL-8 expression. The data reflect gating on monocytes, based on forward and side scattered light signals. The result shown is a representative of two experiments conducted with different PBMC preparations that had similar results.

**Figure 2:**
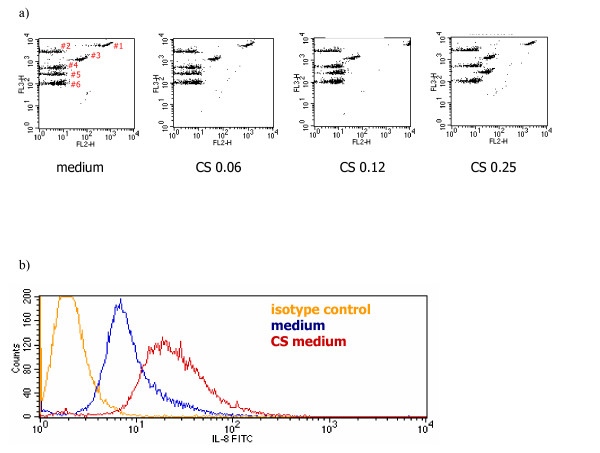
After exposure to CS medium, human monocyte-derived macrophages produce IL-8. a) Macrophages were stimulated overnight with different concentrations of CS medium. Cytometric Bead Array assay was performed to quantify cytokine secretion. Representative dot plots of #1. IL-8, #2. IL-1β, #3. IL-6, #4. IL-10, #5. TNF-α, and #6. IL-12. b) Cells were stimulated with CS medium (OD = 0.03) for 5 h in the presence of GolgiStop™. The cells were stained for intracellular IL-8 expression. The result is a representative of five experiments conducted with different human monocyte-derived macrophage preparations that had similar results.

Because little is known about the activation of primary human cells by CS medium, an exploratory study was performed measuring several inflammatory cytokines which are also known to be involved in COPD. PBMC stimulated by CS medium produced inflammatory cytokines such as high amounts of IL-8 (5 ng/ml) (data not shown). Further experiments showed that monocytes in comparison with neutrophils and lymphocytes are the major source of IL-8 generation in PBMC (Fig. 1A). In addition, high expression of intracellular IL-8 was demonstrated in CD14 positive cells (Fig. 1B). Since these findings suggested that most likely monocytes are the major source of IL-8 production after exposure to CS medium, a human monocyte-derived macrophage culture system was established. Purified monocytes were cultured for 5 days in medium containing GM-CSF and M-CSF to gain macrophages [24]. Macrophages showed responsiveness to CS medium in a dose dependent manner and released pro-inflammatory cytokines e.g. IL-6, IL-8 and TNF-α(Fig. 2A). Intracellular cytokine staining demonstrated high levels of IL-8 expression in human macrophages after 5 hr stimulation with CS medium (Fig. 2B).

### CS medium-induced IL-8 production by human monocyte-derived macrophages is TLR-4 mediated

**Figure 3:**
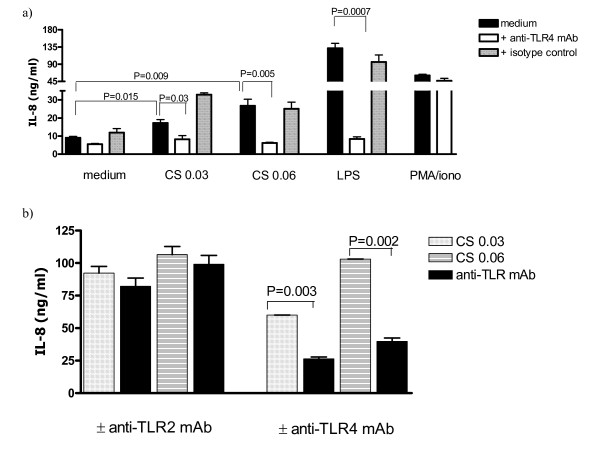
IL-8 production by human macrophages is TLR4 mediated. a) Cells were incubated overnight with anti-TLR4 or isotype control prior to CS medium or LPS or PMA/ionomycin exposure. IL-8 production was quantified using ELISA. The result is a representative of 3–5 experiments conducted with different human monocyte-derived macrophage preparations in which the mean fold increase in IL-8 production at concentration of CS = 0.03 was 1.8 ± 0.31 (n = 5) and the mean percentage of reduction in IL-8 production in the presence of the anti-TLR4 antibody was 66.7 ± 8.6 (n = 5). b) Cells were incubated with anti-TLR2 or anti-TLR4 prior to CS medium exposure and Cytometric Beads Array assay was performed. The result is a representative of 3 experiments conducted with different human monocyte-derived macrophage preparations that had similar results.

CS may contain bacterial endotoxin [27] and many other different inflammatory stimuli. We analyzed the samples for endotoxin biological activity using the Limulus assay. The amount of endotoxin in the applied CS medium was less than 3 pg/ml (data not shown). Then macrophages stimulated with polymyxin bead treated CS medium. The amount of IL-8 release just varied from 21.6 ± 1.02 ng/ml to 19.8 ± 3.6 ng/ml when the medium was treated with polymyxin beads (data not shown). Thereafter, the involvement of TLR2 or TLR4 in CS medium-induced IL-8 production by macrophages was investigated. Pretreatment of macrophages with anti-human TLR4, markedly blocked IL-8 secretion in response to CS medium (Fig. 3A) while no inhibition was observed when the cells were pre-incubated with anti-human TLR2 or mouse IgG2a isotype control (Fig. 3B). Moreover, anti-human TLR4 failed to inhibit PMA/ionomycin-induced IL-8 generation by macrophages (Fig. 3A).

### CS medium-induced signaling pathways in human monocyte-derived macrophages are IRAK and TRF6 mediated

**Figure 4:**
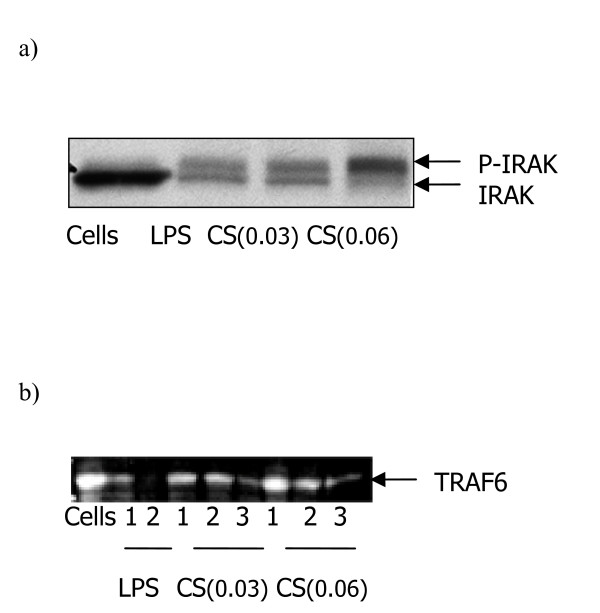
CS medium triggers signaling pathways mediated IRAK and TRAF in human monocyte-derived macrophages. a) Macrophages were treated with CS medium or LPS for 45 minutes and IRAK activation was monitored by western blot analysis. The figure shows autophosphorylation of IRAK (p-IRAK). B) TRAF6 degradation was determined after 1 to 3 hrs CS medium or LPS exposure to macrophages. Cells were lysed and western analysis was performed. The result shown is a representative of two experiments conducted with different human monocyte-derived macrophage preparations that had similar results.

In the TLR-mediated signaling pathways, IRAKs and TRAF6 play critical roles, as demonstrated by analysis in gene targeted mice [28]. Activation of IRAK shown to be the first event downstream of recruitment of the adaptor molecule MYD88 in the TLR4 signaling pathways [29]. CS medium treated macrophages showed IRAK phosphorylation after a 45 minute exposure of CS medium (Figure 4A). TRAF6 is a critical component of TLR4-mediated signaling pathways at level downstream of IRAK [28,30]. Western analysis showed that CS degrades TRAF6 after 1hr exposure to human macrophages and complete degradation achieved after a 3hr-treatment of the cells with the smoke medium (Figure 4B).

### CS medium stimulation of human monocyte-derived macrophages leads to NF-κB activation

**Figure 5:**
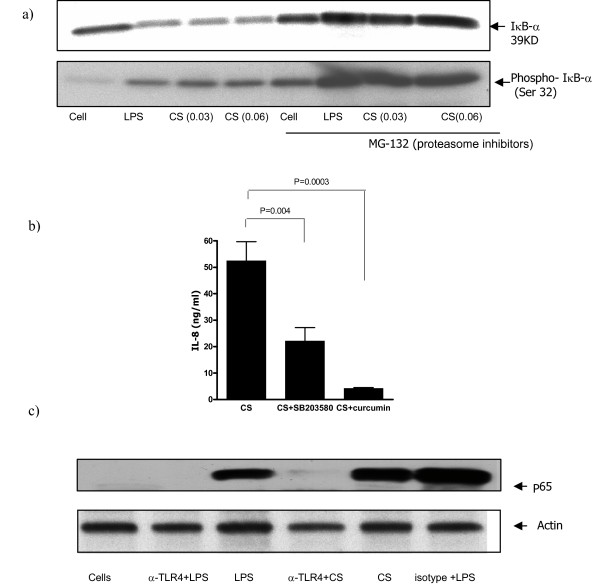
NF-κB involvement in CS medium stimulation of human macrophages for IL-8 production. a). Cells were left in culture medium or incubated with proteasome inhibitor, MG-132, at 10 μM for 1 hr prior to a 45 minute treatment with CS medium or LPS. Macrophages were lysed to determine IκB-α and phosphorylated IκB-α. b). Macrophages were treated for 30 min with SB 203580 (5 μM) and curcumin (25 μM) prior to CS medium exposure. IL-8 production was quantified using ELISA. c). Cells were left in culture medium or incubated with anti-TLR4 or isotype control prior to a 30 minute exposure to CS or LPS. Nuclear proteins were extracted, subjected to 10 % SDS-PAGE, and blotted with P65 Abs. The result shown is a representative of three experiments conducted with different human monocyte-derived macrophage preparations that had similar results.

TLR-mediated signaling pathways via IRAK and TRAF6 leads to NF-κB activation [28]. To investigate whether NF-κB activation is also involved in IL-8 secretion by human monocyte-derived macrophages after CS stimulation, cells were treated with increasing concentrations of CS medium and whole-cell extracts were immunoblotted for phosphorylated IκB. As shown in Figure 5a, an increase in phosphorylated IκB levels was observed after exposure to CS medium. In contrast, stabilized IκB levels was demonstrated as macrophages pretreated with proteasome inhibitor, MG-132, for one hour before exposure to CS (Fig. 5A). Moreover, we examined the involvement of NF-κB in CS-induced IL-8 production by treatment of macrophages for 30 min with NF-κB inhibitor curcumin prior to CS medium exposure (Fig. 5B). We found inhibition of IL-8 release by 85% (from ~52.5 ± 7 ng/ml to ~4.2 ± 0.3 ng/ml) after pretreatment of macrophages with curcumin at concentration of 25 μM. Next, we incubated the cells with p38 MAP kinase inhibitor SB 203580. SB 203580 at concentration of 5 μM inhibited the IL-8 generation by macrophages. The amounts of IL-8 produced and released by the cells were diminished by 42% (from ~52.5 ± 7 ng/ml to ~22.0 ± 5.1 ng/ml). Furthermore, we studied the translocation of NF-κB subunit p65 to the nucleus following CS activation. As shown in Figure 5C an increase in p65 level was detected in the CS stimulated sample. In contrast, p65 level in nuclear extracts was not detected upon anti-TLR4 antibody treatment of the cells prior to CS exposure.

## Discussion

The mechanisms responsible for induction of inflammatory reactions by CS have yet to be elucidated. We used a medium collected from main stream and side stream of CS to stimulate human monocyte-derived macrophages. The present study shows for the first time that macrophages can be stimulated by CS in a dose dependent manner (Figure 1 and 2) to produce cytokines which is mediated by a cascade of TLR4 signaling events (Figure 3).

Because of the high levels of IL-8 generation by cultured macrophages (Figure 2), IL-8 secretion was monitored to study the mechanisms by which CS medium induced inflammatory cytokines. First we investigated whether or not the effect is due to LPS that might be present CS extract? We analyzed the samples for endotoxin biological activity using the Limulus assay. The amount of endotoxin in the applied CS medium was less than 3 pg/ml, which is most likely not enough to trigger the cytokine production by human macrophages. Polymixin B is an antibiotic that contains a cationic cyclopeptide with a fatty acid chain that can neutralize the biological activity of endotoxins by binding to the lipid A portion of the bacterial LPS [31-33]. We exposed the cells to CS medium which has been treated with polymixin beads. Macrophages stimulated with polymyxin bead treated CS medium did not show a significant decrease in the amounts of IL-8 generation (data not shown). The amount of IL-8 release in response to CS medium just varied from 21.6 ± 1.02 ng/ml to 19.8 ± 3.6 ng/ml when the medium was treated with polymyxin beads demonstrating that the effects of CS is not due to LPS presents in the medium.

Since CS extract contains many inflammatory stimuli, two well described TLRs, TLR2 and TLR4 which are expressed in human macrophages, were studied. We found that neutralization of TLR4 but not TLR2 inhibits CS medium-induced IL-8 secretion by human macrophages (Figure 3). The discrepancy can be explained by the recent report suggesting that the functional outcomes of signaling via TLR2 or TLR4 are not equivalent and in spite of their shared capacities to activate the same signaling molecules, different TLRs are capable of activating distinct cellular responses [34].

The possibility of changes in cellular behavior of macrophages after incubation with TLR4 neutralizing antibody was studied. The amounts of IL-8 release after stimulation with PMA/ionomycin was monitored (Figure 3). PMA stimulates PKC and ionomycin increases intracellular calcium [35]. The cytokine production by human monocyte-derived macrophages is modulated by PKC [36]. We pretreated the cells with anti-human TLR4 antibody before CS medium exposure and examined the amounts of IL-8 generation. We demonstrated that macrophages produce IL-8 in response to PMA/ionomycin and the amount of IL-8 release is not affected by TLR4 neutralizing antibody (Figure 3A). Indeed, the same levels of IL-8 production by macrophages following PMA/ionomycin stimulation in the presence or absence of neutralizing antibody suggest that Anti-TLR4 inhibition of CS-induced IL-8 release is not due to cellular damage but blockade of TLR4. Then, TLR4 and its downstream pathways were studied. TLR4 ligation leads to NF-κB activation and signals via IRAK and TRAF [29,30]. Our observations show that the signaling cascade of TLR4 ligation by CS medium involves IRAK-1 phosphorylation (Figure 4A). Additionally, we found that TRAF6 degradation is also involved in the signaling pathways (Figure 4B). Ligation of TLR4 by LPS activates NF-κB and induces production of cytokines in human myeloid cells [37]. Moreover, induced transcriptional activity of NF-κB leads to maximal amount of IL-8 generation (19). We demonstrated increases in phosphorylated IκB-α levels after CS medium stimulation of macrophages (Figure 5A). The proteosome inhibitor MG-132 blocks the degradation of IκB-α [38]. As shown in Figure 5A, an increase in the phospho- IκB-α level was detected in the CS-treated samples (see lanes cells and cells plus CS). In contrast, the samples pretreated with MG-132 did not show such levels of phospho- IκB-α upon CS stimulation. Moreover, the degradation of IκB-α is blocked when the cells were exposed to MG-132 (lanes MG-132). The natural product curcumin is a known inhibitor of activation of NF-κB. Involvement of NF-κB in CS-induced IL-8 production was demonstrated when macrophages were treated with NF-κB inhibitor curcumin prior to CS medium exposure. Curcumin completely blocked the CS induced IL-8 production (Fig. 5B). The anti-inflammatory properties of curcumin and its ability to inhibit the immune response upon exposure to a variety of external stimuli may, at least in part, result from inhibition of the activation of NF-κB by these external signals, since many of the genes that are implicated in the immune/inflammatory response are up-regulated by NF-κB. For example, curcumin inhibits the LPS-induced production of IL-1β and TNF-α which NF-κB is implicated in these signaling pathways [39]. SB 203580 is an inhibitor of p38 MAP kinase. Recently, significant advances in the understanding of signaling pathways, which coordinately regulate IL-8 transcription as well as mRNA stabilization in response to external stimuli, have been made. The maximal IL-8 amounts can only be generated if the resulting mRNA, after NF-κB translocation, is rapidly stabilized by the p38 MAPK pathway[40]. Blocking the p38 MAPK pathway by SB203580 decreased the amount of IL-8 generation suggesting that p38 MAPK pathway involves in the maximal amounts of IL-8 production after CS exposure. More studies are needed to demonstrate that whether the role of p38 MAPK pathway is to stabilize IL-8 mRNA after CS stimulation or the pathway at least partially activates NF-κB activation. It has been demonstrated that SB203580 attenuates lysophosphatidic acid-dependent phosphorylation of I-κB, NF-κB and NF-κB transcription in human bronchial epithelial cells [41]. These findings suggest that SB203580 by itself might be involved in NF-κB activation. The increases in phosphorylated IκB-α levels as well as the dramatic decline in amount of IL-8 secretion by NF-κB inhibitor (see above) showed that NF-κB activation is involved in the pathways of CS stimulation of human macrophages.

The five members of the mammalian NF-κB family, p65 (RelA), RelB, c-Rel, p50/p105 (NF-κB1), and p52/p100 (NF-κB2), exist in unstimulated cells as homo- or heterodimers bound to I-κB family proteins. NF-κB activation leads to the translocation of thetranscription factors from the cytoplasm to the nucleus. [42,43]. We studied the translocation of NF-κB subunit p65 to the nucleus following CS activation. We detected an increase in p65 level in nuclear protein extracts following CS exposure (Figure 5C). Furthermore, no p65 was detected in the nuclear protein extracts where the cells were pre-treated with anti-TLR4 prior to CS medium stimulation (Figure 5C). These findings confirm that CS induces NF-κB translocation to the nucleus and that this can be inhibited by blockade of TLR4.

In conclusion, the results presented here show that the mechanism underlying IL-8 production by human macrophages after CS medium exposure involves activation of TLR4 specific signaling pathways. It has to be stressed at this point that a secreted TLR2 agonist from different bacterial LPS, induced distinct patterns of cytokine production by macrophages [34]. Therefore, we can not rule out the possibility of the ligation of TLR4 by different LPS or bacterial endotoxins present in CS medium. However, these compounds are part of CS extract and must be considered as one of the inflammatory stimuli of CS constitutes which might triggers initial lung inflammation in COPD.

In our study no analysis done to characterize the chemical nature of the activity present in CS medium. For instance, our CS medium may contain reactive oxygen species (ROS) which activates NF-κB [44] and may regulate immune signaling through TLR4. Further studies are needed to address firstly the presence of ROS in our CS medium preparations and secondly the possible ROS interaction with TLR4 signaling.

During the preparation of the manuscript, Droemann et al. [45] proposed a smoke related change in the phenotype of alveolar macrophages demonstrating a reduced expression of TLR2 in smokers and COPD. Although the alteration is restricted to TLR2 expression but supports the hypothesis that COPD pathogenesis might be associated to stimulation of macrophages through TLRs.

Peripheral blood monocyte-derived macrophages are a unique cell type generated in vitro and are an attractive cell model to study the role of macrophages in inflammatory process. However, consideration of how the findings can be linked to the human disease must be given. Indeed, human alveolar macrophages can be employed to further examine the validity of our findings in the context of human disease.

## Conclusion

Increased levels of IL-8 in patients with mild-to-moderate COPD has been demonstrated suggesting that the migration of neutrophils and mononuclear cells from the bronchial wall to the lumen could be increased through IL-8 [3]. Our study suggests that in lungs, macrophage-derived IL-8 after TLR engagement may trigger the recruitment of neutrophils and CD8 positive T cells, both the major effector cells in COPD inflammatory process. The clarification of the mechanisms of macrophage activation by CS through this TLR may offer new insight into the treatment of COPD. In conclusion, our observations suggest a cellular mechanism that links smoking with inflammation in COPD.

## Abbreviations

**COPD**: Chronic Obstructive Pulmonary Disease, **CS**: cigarette smoke, **MDM**: Monocyte-derived macrophages, **BAL**: bronchoalveolar lavage, **TLR**: Toll-like receptors, **MFI: **Mean Fluorescence Intensity

## Competing interests

The author(s) declare that they have no competing interests.

## Authors' contributions

KK conceived of the study, and participated in the design of the study and performed immunoassays, FACS analysis, statistical analysis, and wrote the first draft and final version of the manuscript. HS and EM carried out the ELISAs and biochemical experiments. JJS participated in performing the experiments and took part in critical revision of the manuscript. SHH contributed in performance and plans of the experiments. SJDK initiated the project and participated in the design of the study and critical revision of the article for important intellectual content. FPN participated in the design and coordination of the study. GF conceived of the study, and participated in the design of the study and supervised the project. All authors read and approved the final manuscript.
